# Pathobiology of Cognitive Impairment in Parkinson Disease: Challenges and Outlooks

**DOI:** 10.3390/ijms25010498

**Published:** 2023-12-29

**Authors:** Kurt A. Jellinger

**Affiliations:** Institute of Clinical Neurobiology, Alberichgasse 5/13, A-1150 Vienna, Austria; kurt.jellinger@univie.ac.at; Tel./Fax: +43-1-5266534

**Keywords:** Parkinson disease, cognitive impairment, dementia, neuroimaging, neuropathology, functional connectivities, neuronal network dysfunctions, superimposed pathologies

## Abstract

Cognitive impairment (CI) is a characteristic non-motor feature of Parkinson disease (PD) that poses a severe burden on the patients and caregivers, yet relatively little is known about its pathobiology. Cognitive deficits are evident throughout the course of PD, with around 25% of subtle cognitive decline and mild CI (MCI) at the time of diagnosis and up to 83% of patients developing dementia after 20 years. The heterogeneity of cognitive phenotypes suggests that a common neuropathological process, characterized by progressive degeneration of the dopaminergic striatonigral system and of many other neuronal systems, results not only in structural deficits but also extensive changes of functional neuronal network activities and neurotransmitter dysfunctions. Modern neuroimaging studies revealed multilocular cortical and subcortical atrophies and alterations in intrinsic neuronal connectivities. The decreased functional connectivity (FC) of the default mode network (DMN) in the bilateral prefrontal cortex is affected already before the development of clinical CI and in the absence of structural changes. Longitudinal cognitive decline is associated with frontostriatal and limbic affections, white matter microlesions and changes between multiple functional neuronal networks, including thalamo-insular, frontoparietal and attention networks, the cholinergic forebrain and the noradrenergic system. Superimposed Alzheimer-related (and other concomitant) pathologies due to interactions between α-synuclein, tau-protein and β-amyloid contribute to dementia pathogenesis in both PD and dementia with Lewy bodies (DLB). To further elucidate the interaction of the pathomechanisms responsible for CI in PD, well-designed longitudinal clinico-pathological studies are warranted that are supported by fluid and sophisticated imaging biomarkers as a basis for better early diagnosis and future disease-modifying therapies.

## 1. Introduction

Parkinson disease (PD), the most common movement disorder and the second most common neurodegenerative disease after Alzheimer disease (AD), is characterized by progressive degeneration not only of the dopaminergic striatonigral system, responsible for the core motor symptoms, but also of many other neurological systems and organs. These lesions are due to the widespread intraneuronal and neuritic deposition of phosphorylated α-synuclein (αSyn), forming Lewy bodies (LBs) and Lewy neurites, which are the morphological hallmarks of PD and related synucleinopathies. They are part of multiple pathways in the pathogenesis of PD which include oxidative stress, abnormal proteasome, mitochondrial, synaptic and lysosomal dysfunction, and neuroinflammation, which is related to the spreading of abnormal proteins in cortical and subcortical regions with the subsequent dysregulation of multiple neurotransmitter systems [1,2,3,4,5,6]. The resulting biochemical deficits induce a heterogenous clinical spectrum of motor and non-motor symptoms that contribute to the overall disease burden of this multisystem/organ disorder [7,8,9]. Cognitive impairment (CI) that has been increasingly recognized as an integral part of PD since its historical description by Charcot (1877) [10] shows a complex spectrum ranging from subtle cognitive decline (SCD) and mild cognitive impairment (MCI) to full-blown dementia (PDD), and it can be present already during the prodromal phase of PD [11,12]. SCD is a self-perceived decline in cognitive abilities with normal age-, sex- and education-adjusted performance on standardized cognitive tests indicating adequate cognitive functions [13], although there is currently no consensus on clinical criteria for SCD in PD [14]. Since it is already associated with brain metabolic changes in multiple cortical regions indicating early aberrant pathological changes [15], there is an increasing risk of developing dementia [16]. Parkinson disease with mild cognitive impairment (PD-MCI), a gradual decline in single or multiple cognitive domains [17,18] and a risk factor for PDD [19], is characterized by deficits in at least four cognitive domains (executive and visuospatial abilities, attention and memory), being severe enough to significantly affect routine functions of life [20,21,22,23]. Cognitive decline may occur in presymptomatic stages of PD [24] preceding the onset of dementia by up to 20 years as well as at the time of diagnosis or during the disease process. It has a high variability in severity, rate of progression and type of affected cognitive domains [3,25,26], but its structural and molecular backgrounds are incompletely understood [27,28]. CI has severe consequences even at early disease stages, poses a heavy burden on both patients and caregivers, affects the quality of life and is a risk factor for early mortality [14,29,30,31]. Novel genetic, fluid or imaging markers appear promising in facilitating the diagnosis and staging of CI in PD, and the stabilization or amelioration of cognitive outcomes is a goal of many ongoing clinical trials for disease-modifying treatment options. This article, based on literature research in PubMed and Google Scholar until November 2023, will critically review the currently available data about the epidemiology, essential clinical, neuroimaging, connectivity, morphological substrates, and biomarkers of CI in PD. The relations between PDD and dementia with Lewy bodies (DLB), considered as two heterogenic phenotypes of the LB continuum, will not be discussed in this review, since they have been reviewed recently [32,33,34,35,36,37], including connectivity studies in DLB [38].

## 2. Epidemiology of CI in PD

### 2.1. PDD—General Items

PD patients have a three to five times higher risk of developing CI than people without PD of similar age [25]. PDD has a four to six times increased lifetime incidence rate compared with age-matched controls [39,40]. The current prevalence of dementia is 31.3% (95% CI 20.1–40.1), with incidence rates from 42.6 to 112.5/100,000 person-years [41], indicating that around 10% of a PD population develop dementia per year [42]. The incidence of PDD increases with the duration of the disease from 3% to 30% of individuals followed for 5 years. Its prevalence is between 48% and 78% with a mean of 75% after survival for more than 10 years [43] and 83% after 20 years [40]. The relative risk for developing dementia in incident PD subjects compared to non-PD subjects was 2.47 (1.55–2.39) [39]. Systemic reviews suggest that 3–4% of dementia in the general population would be due to PDD; its estimated prevalence in the population older than 65 years being 0.2–0.5% [44]. The global pooled frequency of PDD is 26.3% with variations according to methodologies (14–55%) [45]. Its cumulated prevalence in PD patients with a mean age of 54–70 years is 17% at 5 years and 83% at 20 years after diagnosis and up to 95% by age 90 years [46]. Of note, there are several challenges in estimating the frequency of cognitive change in PD due to recently standardized diagnostic criteria variations in neuropsychological evaluation and differences in population sampling [47].

### 2.2. PD—Subjective Cognitive Impairment and MCI

Since there are no guidelines for PD-SCD, researchers have classified it differently, and some defined it as subjective CI without objective cognitive decline but poor performance in action naming [48] and an indicator of subsequent CI [49,50]. Its incidence, therefore, varied considerably between 25% and 85% [14,51,52,53,54,55,56].

MCI, a transitional stage between normal cognition and dementia, can occur in the presymptomatic phase of PD; it affects 19–30% of newly diagnosed, untreated (de novo) PD patients and has a frequency ranging from 21% to 70% with a mean of 25.8% [25,57,58,59,60,61]. About 30% of de novo PD patients complain of memory issues and are more likely to develop MCI after 2 years follow-up [50], but other factors, such as anxiety and affective symptoms, may contribute to the progression of cognitive deficits [62,63]. Although the reported estimates of cognitive dysfunction in non-demented PD patients vary between 19 and 55%, CI in non-demented PD is underestimated in practice [64]. At least 20–30% have at least mild cognitive changes even at the time of PD diagnosis [65], increasing to 40–50% after 5 years follow-up [66] and to 75% after survival of more than 10 years [40]. A recent meta-analysis reported a pooled MCI prevalence of 40% [67], while the estimated prevalence of MCI in the general population (age 60–90 years) ranges between 16 and 20% [68].

About 60.5% of PD patients were diagnosed with MCI when comprehensive assessment was performed (MDS criteria level II), while this number was 23.3% when only brief assessment was available (Movement Disorder Society/MDS criteria level I); multiple domain impairment was most frequent (96.2%). In contrast, 42.3% of MCI cases developed dementia at follow-up [69]. Between 20 and 57% of PD patients were affected by MCI within the first 3–5 years after diagnosis [27,70] with a predominance of non-amnestic MCI (naMCI) [71]. MCI is found in 50% of patients with idiopathic REM sleep behavior disorder (RBD), a frequent precursor of PD, and in 73% of PD patients with RBD, but in only 11% of those without RBD [72]. MCI was almost threefold higher in PD patients with RBD compared to those without it (66% vs. 23%; *p* < 0.001), and subjective cognitive decline was reported in 89% of PD + RBD compared to 58% of those without RBD [73]. More recent studies reported a prevalence of MCI level I of 12.8% in the lower risk, 21.9% in the higher risk, and 64% in RBD patients, 66% of which had multidomain level II MCI, particularly attention and memory deficits [74].

The classification of MCI for 12 neuropsychological criteria found 14% PD-MCI using two scores in one domain at two standard deviations below normal scores and 89% PD-MCI with one score in one domain at one standard deviation below normative scores [75]. A multicenter analysis revealed an average prevalence of MCI of 25.8%, 18.9% in an incidental, untreated cohort, and 39.4% in advanced PD. The cognitive phenotype was heterogenous; the most common subtypes were amnestic and non-amnestic MCI (aMCI, naMCI) single domain (11.3% and 8.9%, respectively) [76]. More recent studies reported 69.3% PD-MCI, 16% PD with normal cognition (PD-NC) and 14.66% PDD [77] or 34% PD-MCI with 77.8% impaired in multiple cognitive domains [59].

Among patients with PD-NC, within 3 years, 25% (95% CI 20–30%) converted to PD-MCI and 2% (95% CI 1–7%) converted to PDD, whereas 28% (95% CI 20–37%) reverted back to normal cognitive functions [78]. Meanwhile, 59% of PD patients with persistent MCI within one year developed PDD [18]. The incidence of progression from PD-MCI to PDD was 98.0/1000 person-years with an annual conversion rate of 11% [79]. The PDD converters had higher frequencies of multidomain MCI and amnestic MCI with poorer performance of frontal/executive, memory and language function domains [80]. Cognitive domains with higher frequencies of impairment were found in verbal memory and attention/processing speed, but no significant differences in the prevalence of CI were found during a 5 years follow-up [81].

In conclusion, the incidence and prevalence of CI/dementia is higher in PD, and the development of CI (initially MCI) is accelerated in PD in comparison to the age-matched control population.

## 3. Risk Factors of CI in PD

Risk factors for CI/dementia in PD are old age, lower education, duration of disease and motor symptom severity, in particular predominant rigidity, postural imbalance, and various non-motor symptoms, such as depression, psychosis or hallucinations [39,82,83,84]. Cognitive dysfunction progresses more rapidly in patients with older disease onset, the frequency of MCI and dementia in young-onset patients (50 ± 8 years) being 5% and 10% compared to 25% and 22.3% in older ones (61 ± 8.3 years) (*p* < 0.0001) [85]. Women experience a worse global cognitive decline during the prodromal phase of PD [11]. RBD is a cognitive risk factor across individuals of all ages with recently diagnosed PD [74,86]. Increased homocystein levels in peripheral blood are another risk factor for cognitive decline in PD, although no association was found between the polymorphism of genes involved in homocystein metabolism [87]. Both diabetes and prediabetes also may negatively affect cognitive function in PD, with a significant interaction of diabetes status and age, but not with duration of PD [88]. Gut microbiome composition (reduced short-chain fatty acid-producing bacteria) has been associated with PD-CI [89]. Both increased uric acid levels and altered glomerular filtration rate with lower cerebrospinal fluid (CSF) amyloid-β (Aβ) and αSyn, and higher neurofilament light (NfL) in serum are associated with CI in PD [90,91]. Orthostatic hypotension prevalence is correlated to severity of CI, particularly with a lower performance in executive functions (EFs) [92]. It is a significant risk factor for CI in PD, especially in women and persons not suffering from hypotension [93]. Early hypotension, but not its symptom severity, increased dementia risk in PD patients by 14% while not being associated with other clinical or neuropathological variables [94]. PD patients with freezing of gait (FOG), an important risk factor for CI, exhibit worse global cognition, EF/attention, language memory and visuospatial functions [95,96,97]. Another risk factor for PDD is the overuse of anticholinergic drugs, especially in older PD patients with multimorbidities [98]. The presence of cerebral small vessel disease, often related to diabetes mellitus, hyperhomocysteinemia and hypertension, is an independent risk factor for PD-CI [99]. A review of the wide spectrum of risk factors of PD using the ICD-10 was published recently [100]. On the other hand, there is an association between poor cognitive functioning and risk of increased parkinsonism, including probable PD [101].

## 4. Genetic Factors of PD-CI

Although most cases of PD are sporadic and only approximately 7–10% result from monogenic cause, either autosomal dominant or recessive [102,103], nearly all PD is genetically influenced. More than 100 genes or genetic loci have been identified, and most cases likely arise from interactions among many common or rare genetic variants [4]. CI has been reported in all monogenic PD forms with variable rates. Currently, there are seven well-described genes that increase the risk for PD with variable rates of penetrance: in addition to *SNCA*, *LRRK2*, VPS35, PRKN, PINK1, *DJ1* and the glucocerebrosidase gene *GBA* [104], there are PD risk variants, including *PITX3*, *TMEM106B*, *SNCA* Rep1, *APOE* ε4, *COMT* and *MAPT* H1/H1 with respective relationships to cognition [105]. Furthermore, seven upregulated genes, namely *SNAP25*, *GRIN2A*, *GABRG2*, *GABRA1*, *GRIA1*, *SLC17A6*, and *SYN1*, have been identified, which are significantly correlated with PDD [106]. *GBA* mutations have a negative impact on cognition, whereas *LRRK2*-associated disease may have a milder cognitive phenotype, the others having a potential effect on cognitive outcome [107]. GBA-PD is associated with faster motor and cognitive decline, especially with impaired visuospatial/EFs [108,109], whereas the rs12411216 variant of *GBA* was significantly associated with PD-MCI [110]. *PARK16* rs6679073 carriers were less likely to develop MCI compared to non-carriers in a 4-year follow-up study, suggesting that this variant may have a neuroprotective effect on cognitive functions [111]. Longitudinal cognitive decline in patients with GBA-PD was more severe than in those with LRRK2/GBA-PD, which more closely resembled LRRK2-PD. This supports the notion of a dominant association in individuals who carry both alleles and raises the possibility of an *LRRK2* and *GBA* interaction, although its biological basis is not clear [112]. *APOE* ε4 has been identified as a major risk factor for cognitive progression in PD [113]. It has an age-dependent effect on decline in global cognition as well as in most cognitive domains [114,115]. Carriers of *APOE* ε4 and GBA mutations had faster cognitive decline and were at higher risk of progression to dementia [116], whereas no significant gene-related effects were observed for *MAPT* and *SNCA* rs356219 [117]. In PD patients, aquaporin-4 polymorphism (rs162009) was associated with slower dementia conversion and better performance in semantic fluency, while subtype rs68006382 was associated with faster progression to MCI, worse performance in semantic fluency and other cognitive domains. Genetic variations of aquaporin-4 may contribute to an altered rate of cognitive decline probably due to subsequent alterations of glymphatic efficacy, which is a sleep-enhanced brain waste clearance system [118]. Finally, a possible role of glial cell line-derived neurotrophic factor (*GDNF*) on CI in PD has been suggested, although α- and β-GDNF are not used for predicting CI in PD [119].

In conclusion, recent genetic studies have shown that at least seven well-described genes and a large number of variants are important risk factors for cognitive decline in PD; linking genetic profiles to cognitive outcomes may have an important clinical impact.

## 5. Development and Clinical Profile of CI in PD

Cognitive changes in PD manifest early and are more heterogenous than previously appreciated [64,65]. Their onset is insidious and the evolution is progressive with a mean annual Mini-Mental State Examination (MMSE) decline of approximately 2.3 points [120]. However, some PD patients may exhibit early cognitive deficits that will not evolve into PDD or persist over an extended period [121]. PD-MCI may begin before the motor symptoms [122] and even 5 years before clinical diagnosis of PDD [123]. Males usually show a more rapid progression of CI than females [124], in particular during the pre-diagnostic phase that exceeds rates associated with normal aging [125].

The mean time from onset of PD to severe CI/dementia is approximately 10 to 15 years, although there is wide individual variance [39], and even in the absence of full-blown dementia, 30–67% of patients with advanced PD show clinically moderate CI [126].

MCI, according to current clinical criteria, includes amnestic and non-amnestic types (aMCI and naMCI) [127], which may involve single or multiple cognitive domains. The latter was observed in 99.8% of the PD-MCI patients [59]. The heterogeneity of early de novo PD-MCI suggested the existence of three distinct memory phenotypes: cluster A (65.9%) included memory unimpaired patients, cluster B (23.2%) included those with mild episodic memory disorder (prefrontal executive-dependent phenotype), and cluster C (10.9%) included those with severe episodic disorder, which is related to the co-occurrence of hippocampal-dependent deficits with premotor executive memory dysfunctions. These phenotypes did not differ in terms of motor and other non-motor features, but the attention/executive deficits progressively increased from cluster A to cluster C, which had worse quality of life compared to others. Brain structural imaging correlates substantiated this cluster model [128].

The involvement of the cognitive domains of EF, which includes attention and working memory, is most prominent in early PD [25,129,130], while visuospatial function and global cognitive deficits occur in the mid-stage of the disease [131,132,133]. While deficits in EFs and visuospatial skills were apparent in PD patients compared to controls, many aspects of cognition remained intact [134]. Patients with poor baseline visual performance showed greater decreases in cognition over time and predicted a greater probability of dementia, whereas the retinal thickness of the ganglion cell-inner plexiform layer did not predict cognitive decline [135]. The cognitive scores of the frontal/EF domain contribute the most to predicting dementia [136].

A three-stage clinical sequence related to cognition has been proposed for PD patients, with SCD as the prodromal phase, which is followed by MCI and finally leading to dementia [15,137,138]. Follow-up studies showed a higher risk of developing PD-MCI and PDD for patients with PD-SCD compared to those without SCD at baseline [49,50,53,137]. Lower baseline attention and language scores were associated with progression to MCI, whereas higher baseline scores in all cognitive domains except EF were associated with clinical and psychometric reversion to “normal” cognition [139]. However, an increased risk of PDD conversion persists even in these reverters [78]. PD-MCI patients showed a specific pattern of language dysfunctions [48], while PD-MCI converters had more severe cognitive deficits in frontal EF, immediate verbal memory and visual recognition memory compared to non-converters [140,141]. A meta-analysis of cognitive outcome and prediction in PD-MCI conversion to PDD was made recently [142].

The Movement Disorder Society Task Force (MDSTF) has delineated the following diagnostic criteria for PD-MCI [17,143]: characterization of the clinical syndrome (inclusion criteria), gradual decline in the context of established PD, cognitive ability and deficits in neuropsychological testing or a scale of global cognitive deficits not sufficient to interfere with functional independence; exclusion criteria (PDD, other explanation for CI and PD-associated co-morbidities). The MDSTF has also suggested diagnostic criteria for PDD [20,144] with these most important cognitive features (see [33,37]).

The impairment of more than one cognitive domain, e.g., executive dysfunction, attention, verbal fluency impairment, both phonetic and semantic, is more pronounced in early and moderate stages of PD, executive dysfunction being the most pronounced. Memory complaints are the presenting cognitive problem in 67% of patients with PDD, which affect short-term memory, verbal, non-verbal and visual domains. Construction, praxis and visuospatial functions are more severely impaired than in AD, but language deficits and aphasia are less severe. Fluctuating cognition is present but less often than in DLB.

The nosological classification of PDD was based on the DSM-IV criteria [145], while the MDSTF defined two levels of diagnostic certainty: possible and probable [20]. Dementia was defined as a syndrome of insidious onset and the progressive decline of cognition and functional capacity from a premorbid level, which is not attributable to motor and autonomic symptoms. A study of diagnostic step I allowing the diagnosis of possible PDD showed a sensitivity and specificity of 78% and 95%, respectively, with a positive-predictive value of 70% and a negative predictive value of 97%, indicating an accuracy of 93.4% [146], while the DSM-IV criteria failed to identify 22% of patients fulfilling MDSTF criteria. After the MDS proposals, great advances have occurred in the field of cognitive assessment in PDD, although many studies concluded that the accuracy of these instruments was not entirely satisfactory [147,148,149,150]. A critical point was that these guidelines did not address limitations inherent to populations with low levels of formal education. Since education is a major factor underlying cognitive performance, a revision of the current guidelines appears necessary considering differences among populations with better scores in cognition [151].

## 6. Neuroimaging Findings in CI in PD (See Supplement Table S1 [152,153,154,155,156,157,158,159,160,161,162,163,164,165,166,167,168,169,170,171,172,173,174,175,176,177,178,179,180,181,182,183,184,185,186,187,188,189,190,191,192,193,194,195,196,197,198,199,200,201,202,203,204,205,206,207,208,209,210,211,212,213,214,215])

Although there is a continuum from PD-NC via SCD to PD-MCI to PDD, it appears of interest to describe the essential neuroimaging findings for these cognitive stages separately.

### 6.1. Gray Matter Changes in PD-MCI

The clinical heterogeneity of PD-MCI is reflected in the variability of structural imaging findings, but the identification of a structural signature of this cognitive deficit remains challenging [42]. Structural atrophy may not be associated with any cognitive domain with the exception of visuospatial measures in primary sensory and motor cortices [216]. In PD-NC patients, structural neuroimaging (MRI) may be unchanged or shows mild diffuse brain atrophy with slight changes of the medial temporal lobes [152,153]. Compared to a control group, PD-NC individuals suffered from gray matter (GM) atrophy mainly in prefrontal and limbic lobes and left temporal gyrus [156]. Voxel-based morphometry (VBM) in PD patients with SCD or subjective memory complaints revealed reduced GM intensities in anterior cingulate and right parietal lobe [55,217] and mild thinning of the midsagittal corpus callosum [218]. Mild atrophy affected the orbitofrontal regions, left superior parietal lobule, and more widespread limbic and fronto-parietal cortices [158]. PD-MCI patients showed baseline thalamus atrophy and progressive atrophy in the thalamus, caudate nucleus, presubiculum, and cornu ammonis, which was associated with executive and memory dysfunctions [173]. Cortical thinning in parietotemporal regions but also total GM volume reduction and ventricular enlargement were associated with CI in executive, visuospatial/perceptual and memory domains [159]. GM reduction in temporal, parietal and occipital regions was found in PD individuals with aMCI and to a lesser degree, naMCI, with executive MCI in frontal and striatal, and in those with non-executive MCI, in posterior regions [160]. PD patients with cognitive decline after 18 months, compared to those with stable condition at baseline, had lower cortical thickness within the prefrontal, medial and lateral temporal cortices [162]. PD-MCI showed a significant enlargement of bilateral temporal and left frontal lateral ventricles [154]. After 2 years of follow-up in PD-NC patients, those with a greater third ventricle width developed CI [155].

The reduction in GM density in superior frontal cortex and cerebellum was related to cognitive performance in early PD-MCI [163]. PD-NC patients converting to MCI showed cortical thinning in medial and superior frontal, inferior temporal, cingulate and supramarginal gyri, whereas those with stable normal cognition suffered from progressive cortical thinning mainly in parietal and occipital regions [167].

PD-MCI patients showed reduced gray matter volume (GMV) in the temporal and parietal cortex, putamen, amygdala, hippocampus and cerebellum [166]. Global cognitive dysfunction (mainly non-memory related) was associated with atrophy in the frontal lobe, left middle and inferior temporal gyrus or in the left superior temporal, right fusiform and bilateral lingual regions [219]. A meta-analysis reported higher GM atrophy in the bilateral prefrontal cortex, left insula and angula gyrus, right supramarginal gyrus and midcingulate cortex in the PD-MCI cohort [164], while another meta-analysis reported GM atrophy in the left anterior insula, left inferior and orbital frontal gyrus [168]. The atrophy of limbic lobes was associated with impaired memory, frontal lobe atrophy and frontostriatal metrics with attention/executive decline [156,161].

Early drug-naive PD-MCI showed atrophy of the right entorhinal cortex [220]. Morphometric studies in early PD-MCI patients showed reduced limbic subcortical structures [221]; the hippocampal subfields subiculum, presubiculum, CA1 and fimbria were strongly correlated with CI, subfields GC-DG and fimbria being sensitive in detecting early stage of CI, while the left parasubiculum and presubiculum predicted conversion from PD-NC to PD-MCI [171]. A smaller CA1 region and reduced hippocampal–amygdaloid transition area volume were observed [169]. A 4-year follow-up study reported that both PD-NC and MCI patients showed a more severe decline in anterior and posterior hippocampus volume with a significant reduction in the bilateral CA4 subregion, other hippocampal subfields and the right presubiculum [170]. A statistically significant difference was observed for the raw hippocampal volumes between PD-MCI and PD-NC [222]; a low baseline hippocampal volume and fornix fractional anisotrophy (FA) were associated with faster cognitive decline [161]. Thus, involvement of the temporo-parietal cortex, lateral ventricular enlargement and selective vulnerability of the hippocampus could be structural biomarkers for conversion to PD-MCI [154,157,223,224].

The volumetric distribution of the subcortical structures (caudate nucleus, putamen, thalamus and amygdala) in PD-MCI exhibited an overlap with the PD-NC group due to a lack of spatial specificity in their atrophy levels, but it was able to predict CI [225]. A high free water fraction in the dorsal caudate nucleus and bilateral nucleus basalis of Meynert (NBM) was associated with cognitive decline across several domains except memory [172]. Cortical microstructural changes (increased intracortical diffusivity) in the absence of cortical thinning were associated with cognitive and psychiatric symptoms [226].

### 6.2. White Matter Lesions in PD-MCI

White matter (WM) damage often precedes GM atrophy, the cumulative burden of WM lesions correlating with CI in PD cases without co-pathologies [174]. Prominent WM changes were observed in the earliest stages of PD with intact GMV [178,227,228], and the white matter hyperintensity (WMH) burden differed from that in PD-NC [229]. Specific brain regions, including periventricular and deep WM, had significantly higher WMH load in the PD-MCI than in the PD-NC group [175]. Total and periventricular WMHs at baseline predicted a decline in global cognition, while the total WMH burden predicted the decline of EFs (both *p* < 0.05). Occipital WMH volumes predicted declines in global cognition, visuomotor attention and visuospatial memory, while WMH volumes at baseline did not predict motor decline [176].

De novo PD subjects with high baseline WMH had significantly greater cognitive decline than those with low WMH load [230], and early episodic memory impairment was related to WMH, mainly in the prefrontal and temporal lobes [177]. WMH volume, changing over time, was associated with an impairment of global cognition, EF and language, whereas WM microstructural lesions did not change significantly [231]. However, a reduction in WM volume was not a consistent finding in PD-MCI compared with healthy controls (HCs) [232,233,234], and WMH often did not impact cognitive function in newly diagnosed PD [235], while in other studies, cognitive deficits were not associated with WMH but with neuronal deficits [236]. This suggests that for some phenotypes of MCI, WM lesions may not be prominent in early stages of PD [42]. On the other hand, microstructural damage in the major motor and associative WM tracts was present and progressed even in early phases of PD [237]. PD-MCI had similar but more severe and widespread WM degeneration in commissural fibers and association projections than PD-NC. The FA of the anterior part of right inferior fronto-occipital fasciculus was positively correlated with the Montreal Cognitive Assessment scale, EF and visuospatial function, while the mean diffusivity of the left superior longitudinal fasciculus was negatively correlated. Thus, regional tract-specific microstructural degeneration, especially in association fibers, is a reliable indicator of PD-MCI [185]. Tract-based FA values decreased in the bilateral corticospinal tract, anterior and posterior cingulum, fornix, bilateral superior thalamic radiation, corpus callosum, bilateral superior and inferior longitudinal fasciculus, superior and inferior fronto-occipital fascicles, and bilateral parieto-occipital tract [181]. Widespread frontostriatal WM tract FA reduction was seen in PD-CI [184]. WM abnormalities, associated with significantly lower neurite density and orientation dispersion in the prefrontal region, the cingulum bundle and in thalamo-frontal tracts, were related to cognitive decline [58,182].

WM abnormalities appeared to be widespread [42], involving multiple brain regions with heterogenous patterns and abnormal diffusivity variables in WM adjacent to cortices and limbic substructures [238]. More comprehensive information on WM changes was obtained by combining diffusion tensor imaging (DTI) indices, with an accuracy of 91.67% and sensitivity of 92.86%, thus achieving the best classification in this data set [217].

The involvement of the corpus callosum, cingulum and major association tracts in PD-MCI but not in PD-NC [157,178,179] as well as increased hyperintensity in frontal and interhemispheric WM (genu and body of corpus callosum) were seen [178,180,239]. Thinning of the corpus callosum was correlated with the thickness of the left orbitofrontal cortex in PD-MCI [218]. The corpus callosum and cingulum bundle showed the same trend to decline with cognitive dysfunction [240]. MCI in early PD stages was associated with poorer olfactory function and disordered WM integrity in the anterior olfactory structures [183]. The bilateral cortico-amygdaloid transition area (CAT) and sectors superficial cortex-like region (sCLR) volumes of PD-MCI and PDD patients were less than those of PD-NC and HC ones. The left CAT and sCLR volumes discriminated CI in PD and were associated with hyposmia [241].

In conclusion, GM changes in PD-MCI involve prefrontal areas and hippocampal subregions, WM lesions being more typically observed in the corpus callosum and limbic structures, while WMH burdens in periventricular and deep areas are widespread in early stages of CI in PD, indicating that the impairment of multiple GM and WM may be important causes of cognitive dysfunction in PD-MCI.

### 6.3. Neuroimaging Findings in Converters to PDD (see Supplement Table S2)

In comparison to PD-MCI patients who did not progress to PDD, the converters showed lower GM densities in prefrontal areas, the left insular cortex, the insula, and the bilateral caudate nucleus and lesser cortical thickness extending from frontotemporal cortices to posterior cortical areas [140,167,242]. They suffered faster rate of GM thinning in temporal and medial occipital lobes, limbic subcortical structures [221] and progressive atrophy in frontal lobes [243]. Their cortex was thinner than that of non-converters, bilaterally in the frontal, insula and left middle temporal areas, displaying a more widespread pattern [244]. While PD-MCI patients showed decreased cortical thickness in the left superior temporal, insula, right fusiform and bilateral lingual regions, in those converting to PDD, widespread cortical thinning was detected, including in the left superior temporal gyrus, insula and bilateral fusiform areas [219].

A progressive atrophy of basal ganglia after one year follow-up and widespread cortical thinning over 3–6 years were observed [237]. The PD-MCI converters had lower cortical thickness within the prefrontal, medial and lateral temporal regions compared with the stable group [162], as well as larger WMH volume and higher hyperintensity scales than non-converters, demonstrating that the WMH burden is related to ongoing decline in frontal-lobe-based cognitive performance [245].

### 6.4. Gray Matter Lesions in PDD (See Supplement Table S3)

GM atrophy in PDD was more severe and extensive than in PD-MCI [166]. Whole brain atrophy showed an increase of 1.12% in PDD patients compared to 0.31% in the non-demented group and 0.34% in healthy age-matched controls. Decreased GM volume in PDD patients in the temporo-parietal region was confirmed by subsequent studies [42,224]. Atrophy affected the bilateral temporal gyrus, posterior cingulate and left cingulate gyrus, right parahippocampal lobe, hippocampus, right cuneus and precuneus, left inferior frontal gyrus and left insular lobe [246,247], with unilateral insular involvement in PD-MCI extending to bilateral insular affection in PDD [164]. Bilateral frontal atrophy [248], basal forebrain atrophy [249,250], and decreased volume of the claustrum were associated with cognitive decline [251,252]. Meta-analysis showed consistent GM loss in bilateral medial temporal lobes and striatum [253]. PDD had lower hippocampal volumes than HCs without exhibiting any differences from PD-MCI [254]. Early-onset PDD patients exhibited more severe atrophy in the left anterior cingulate and right inferior temporal gyrus associated with a significant volume reduction in substantia innominata [255]. Memory impairment was associated with frontal and hippocampal diffusivity also in the left cingulum [180,256,257]. There was a positive correlation between MMSE scores and cortical thickness in the anterior temporal, dorsolateral prefrontal, posterior cingulate, temporal fusiform and occipitotemporal cortices. Discriminant analysis showed that mean cortical thickness and hippocampal volume had 80% accuracy in identifying PDD patients, which could be characterized by a specific pattern of cortical thinning [258]. Thalamic dorsomedial nucleus free water (FW) correlated with the Montreal Cognitive Assessment scale changes over both 1 and 3 years, respectively, as did FW changes in the NBM, and baseline hippocampal FW was also associated with CI at 3 years [259].

Cluster analysis of multimodal imaging data identified three PD subtypes based in the prominent GM patterns: one group with widespread cortical and subcortical GMV and WM FA reduction, associated with considerable cognitive deficits, a second group with cortical atrophy limited to orbitofrontal and temporal regions with specific neuropsychiatric impairment, and a third subtype without increased detectable GM atrophy, lack of considerable CI and earlier disease onset [260].

Visual impairment in PD, along with a heterogenous profile of CI, contributed to the development of FOG that was due to impairment of executive control [261] and decreased contrast sensitivity related to volume decrease in the cuneus, the lingual and posterior cingulate gyrus, the superior parietal lobe and middle frontal gyrus, and the central areas of the dorsal and ventral visual information system [262]. FOG converters had reduced regional homogeneity in the bilateral medial superior frontal gyrus, which was negatively correlated with the postural instability and gait difficulty score [263].

### 6.5. White Matter Lesions in PDD

PDD suffered a significantly higher burden of WMH, especially deeper WMH, than PD-MCI [229,264]. WM abnormalities were widespread [42], involving multiple brain regions with a heterogenous pattern of abnormal diffusivity adjacent to cortical and limbic areas [238]. Thinning of the corpus callosum and microstructural WM abnormalities were associated with changes in the orbitofrontal cortex and contributed to CI by disrupting information transfer across interhemispheric and callosal-cortical projections [218,265]. The strongest of DTI changes occurred in the most anterior and posterior callosal segments, reflecting fronto-striatal and posterior cortical type cognitive deficits [265]. The corpus callosum, cingulum bundle and corticospinal tract contributed to decline of cognitive function [240]. The PDD group showed decreased FA and/or mean diffusivity increase in bilateral cingulate tract [266,267], in genu of corpus callosum [268] and hippocampus [269].

In summary, GM changes in PDD predominantly involve temporal regions including hippocampus, frontal and parietal cortex and subcortical areas including thalamus and NBM, while WM lesions predominantly involve the corpus callosum and cingulate gyrus, inducing dysfunction of cortico-cortical and cortico-subcortical networks [270].

## 7. Brain Network Studies

### 7.1. Early PD and PD-MCI

**Figure 1:**
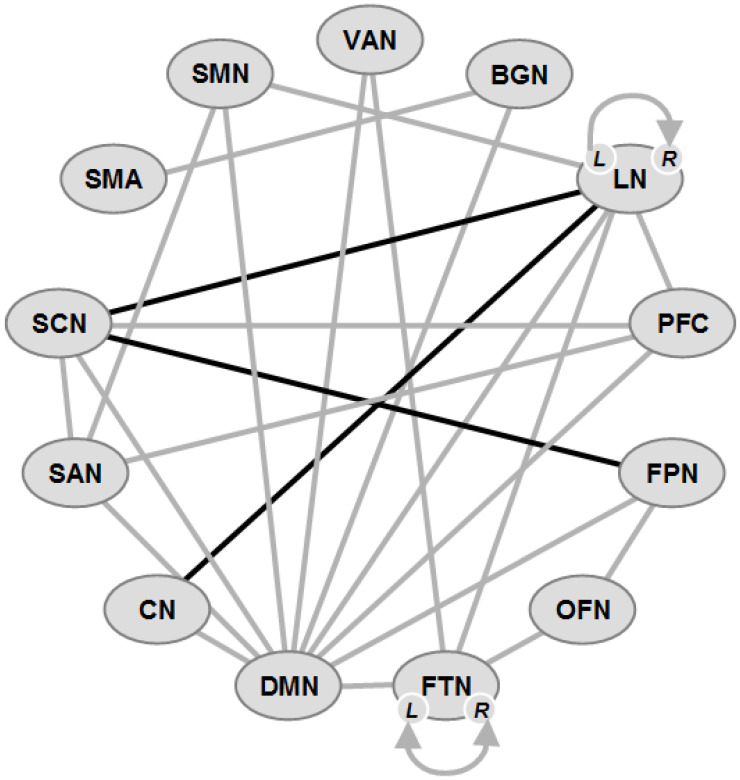
Schematic overview of functional connectivity in some major networks in Parkinson disease with mild cognitive impairment (PD-MCI). Gray lines: hypocommunication, black lines: hypercommunication. L, R: left, right hemisphere. Network abbreviations: VAN: ventral attention; BGN: basal ganglia; LN: limbic; PFC: prefrontal cortex; FPN: frontoparietal; OFN: orbitofrontal; FTN: frontotemporal; DMN: default mode; CN: cerebellar; SAN: salience; SCN: subcortical; SMA: supplementary motor area; SMN: sensorimotor.

Preliminary analyses revealed reduced functional connectivity (FC) in both PD-NC and PD-MCI groups compared with HCs. The PD-MCI group had a significantly decreased FC within the default mode network (DMN), mainly between the hippocampus and inferior frontal gyrus, posterior cingulate cortex and posterior parietal lobule, and between the anterior temporal lobe and inferior frontal gyrus, as well as a significantly decreased FC in the middle frontal and middle temporal gyri. Decreased FC within the DMN between the anterior temporal lobe and inferior frontal gyrus was positively correlated with attention/working performance, while between the hippocampus and inferior frontal gyrus, it was positively correlated with memory function. The PD-MCI group further showed a significant reduction in FC between the DMN precentral, middle temporal gyrus, insula, anterior parietal lobule and middle frontal cortex [193] (Figure 1).

In PD patients with probable RBD, widespread abnormal FC within the most relevant neurocognitive networks, in particular between the left and right frontoparietal network (FPN), the ventrolateral and dorsolateral prefrontal cortex, and between the dorsolateral prefrontal cortex and inferior frontal gyrus, was present [194]. These and other FC changes were already detectable in drug-naive PD patients, even in the absence of clinically overt CI, namely abnormal intrinsic FC within the DMN, executive control network and salience network (SAN), and functional decoupling between the left and right FPN, potentially leading to impairment in cognitive processing and integration or to fronto-striatal maladaptive compensatory mechanisms [195]. PD-NC and PD-MCI patients showed decreased DMN connectivity of the bilateral prefrontal cortex within the FPN, which correlated with some cognitive parameters but not with clinical variables. This suggested that altered DMN connectivity characterizes PD patients, regardless of cognitive status, whereas a functional disconnection of the FPN was associated with MCI in the absence of detectable structural changes [196]. Disruption of the network between the bilateral superior medial frontal gyrus and anterior/middle cingulate cortex appeared relevant for self-awareness of cognition and error processes [201]. In early PD with MCI, a subnetwork predominantly linking temporo-parietal-occipital lobes decreased in both expression and flexibility, reflecting the degree of CI [271].

Disruption of the WM connectivity in frontal and posterior regions was associated with early CI development [80,202]. While PD-MCI patients showed decreased cortical thickness in the right lingual region, widespread changes in the left superior temporal, left fusiform, right insula and right fusiform areas resulting in decreased FC between these affected regions, and dysfunction of resting-state FC, contributed to cognitive decline in PD [219].

Network-based statistics analysis revealed decreased FC in the sensorimotor network (SMN) and DMN, and increased connections in the SAN and the FPN, indicating that the topological organization of GM networks was disrupted in early-stage PD [198], in which altered FC involved the FPN. Similar connections were involved in the DMN and cerebellar network (CN) in both PD-NC and PD-MCI, to a greater extent in the latter, indicating that DMN and CN disruption characterize PD-MCI [197].

Resting-state functional MRI (rsfMRI) analysis in PD-MCI compared to PD-NC revealed altered FC in the right frontal and bilateral parietal areas, which affected broader areas after longer disease duration, while decreased FC in the right frontal area was correlated with shorter disease duration. Decreased FC between substantia innominata, which is significantly correlated with cognitive performance, and the frontal area may be associated with early-onset MCI, suggesting that cholinergic deficits in frontal brain areas might play an important role in the acceleration of cognitive decline in PD [204,255]. Disrupted topology in high-order functional connectivity—FPN, visual, SMN and subcortical networks (in the precuneus, putamen, lingual and supramarginal gyrus, motor area, postcentral and inferior occipital gyrus)—were identified prior to clinical symptoms of CI [205].

rsfMRI analyses revealed abnormally increased and decreased amplitude of low-frequency intrinsic fluctuations (ALFF) and regional homogeneity (ReHo) in PD-NC individuals within the DMN (posterior cingulate, inferior parietal, entorhinal and somatosensory cortex, parahippocampus, basal ganglia and posterior cerebellar lobule VII), which mediates cognition [206]. The ReHo value in the DMN was closely related to PD cognitive function, and the DMN was affected before the development of CI and continuously deteriorated with disease progression [207].

PD-MCI was associated with reduced FC of the mediodorsal thalamus with the paracingulate cortex and breakdown in the connectivity of the mediodorsal thalamus with the para- and posterior cingulate cortex, respectively, while there was an increased FC of the mediodorsal thalamus and posterior cingulate cortex (anterior cingulate, dorsolateral prefrontal, and dorsal posterior parietal cortex) [200]. FOG converters exhibited diminished FC in the basal ganglia, limbic area and cognitive control cortex [263], and they changed subthalamic nucleus activity, causing FOG [272].

PD-MCI patients further showed decreased FC between the striatal networks. This was partly due to atrophy within the SAN. The seed analysis detected relations between higher MCI scores and lower connectivity between the left caudate head, dorsal anterior cingulate and left middle frontal cortex as well as between the right caudate head and anterior cingulate cortex, precuneus and left supramarginal gyrus, but there was increased FC to the left hippocampus and right cerebellar hemisphere [192].

In conclusion, both early and PD-MCI are characterized by reduced FC in the DMN and SAN, the inferior frontal and anterior temporal cortex, the left and right FPN, and the bilateral superior medial frontal cortices, with increased FC in the SAN and FPN, but there was a breakdown of the connection between the mediodorsal thalamus and the cingulate cortex, as well as the striatal network and its cortical connections, indicating a dysfunction of multiple attentional, cognitive control and other essential networks.

### 7.2. Connectivity Changes in PDD (Figure 2)

High-level cognitive processes are related to intrinsic functional brain networks, specifically the DMN, FPN, and SAN, with the SAN playing a role in modulating DMN and FPN activity [273], and a link between SAN and DMN related to cognitive test scores. The SAN is composed of the midcingulate cortex and insula and is integrating responses to salient stimuli [274]; its control over the DMN and FPN is dysregulated in PD and aging [275,276]. The SAN is a key neural substrate of CI in PD [277]. The basal ganglia network (BGN) is also important, because dopamine deficiency can alter functional brain networks [278,279,280]. However, the reliability of SAN dysfunction and its relationship to other brain networks as well as BGN connectivity with other cortical networks (DMN, FPN and SAN) and their impact on CI in PD are unclear [277,281].

Large-scale connectivity studies found that SMNs decreased their FC in the continuum of PD-associated CI [282]. Disturbed FC was associated with attention, EF, language, memory, learning, and visuospatial and global cognition in the bilateral hippocampus, left putamen, olfactory cortex and bilateral anterior temporal poles, suggesting that the cognitive domain-specific networks are distinct from each other and specific for different cognitive phenotypes, which may be related to specific changes in both FC and brain network topology [216,283,284].

Reduced cognitive performance was associated with FC in the dorsal insular cortex with the DMN [285]. Tracts between the dorsal anterior insular cortex and anterior cingulate cortex showing lower FA and higher diffusivity were related with lower EF and working memory, indicating structural damage in the dorsal limb of the SAN due to the loss of an interconnecting anterior insular and anterior cingulate cortex [286]. The FC between two distinct DMN systems (left-to-right hippocampus) was associated with global and domain-specific CI, while that from the medial prefrontal cortex to the posterior cingulate cortex was related to global and episodic memory impairment, indicating a functionally distinct role of the hippocampal subsystems within the DMN resting state network [287]. Reduced hippocampal FA associated with disrupted cortical FC [288,289], frontal cortical disruption and altered temporal FC were associated with global cognitive decline [269,290]. The severity of EF was correlated with lower dynamic FC between deep gray matter regions (DGMs) and the FPN, declining EF with increasing static DGM-FPN connectivity, and changes in the dorsal attention network [291]. Connectivity between the middle (associative) striatum and precuneus negatively correlated with EFs in PD [292]. Frontal/executive, visual memory/visuospatial, and attention/working memory functions were related to disrupted WM connectivity in the frontal and posterior cortical regions [202].

A decreased claustral structural network, linked to cognitive dysfunctions [293,294], attention or spatial navigation [295], memory and executive task alterations in PD [251], showed significantly decreased FC between the left claustrum and the sensorimotor cortical regions including the postcentral gyrus [296].

Recent rsfMRI studies, using the Parkinson’s Progression Markers Initiative (PPMI), a high-quality database of a large number of PD patients, reported several important findings. (1) There was reduced intra-FPN FC with alterations on posterior parietal nodes in the FPN in PD-CI [277], which was possibly due to reduced parietal metabolism [297], whereas there was no evidence of altered SAN-FPN or DMN-FPN connectivity predicting CI [196,298]. (2) PD-CI patients had dysfunctional SAN-BGN and SAN-DMN FC [277]. The SAN, according to some, included the left and right prefrontal cortex [277,299,300], whereas others did not [274,298,301]. These findings indicate a link between SAN FC with other large-scale brain networks and CI in PD, suggesting that positive FC between these networks is necessary for the SAN to disengage the DMN during cognitive control. Confirming reduced SAN-DMN FC with CI in PD [191,275], they support important relationships between intra-FPN, SAN-BGN and SAN-DMN FC and cognition in PD.

**Figure 2:**
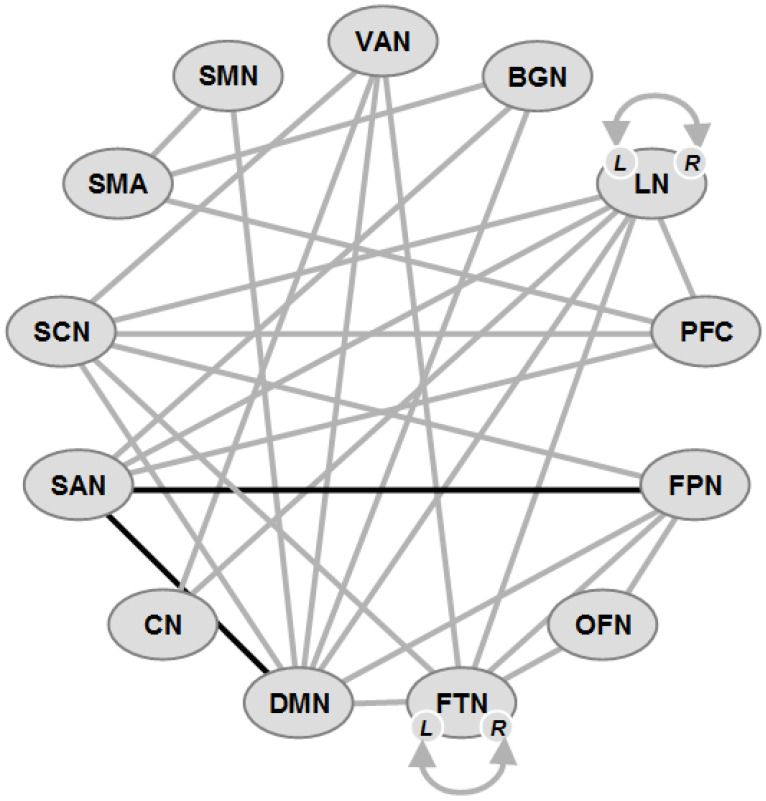
Schematic overview of functional connectivity in some major networks in Parkinson disease dementia (PDD). Gray lines: hypocommunication, black lines: hypercommunication. L, R: left, right hemisphere. Network abbreviations: VAN: ventral attention; BGN: basal ganglia; LN: limbic; PFC: prefrontal cortex; FPN: frontoparietal; OFN: orbitofrontal; FTN: frontotemporal; DMN: default mode; CN: cerebellar; SAN: salience; SCN: subcortical; SMA: supplementary motor area; SMN: sensorimotor.

In conclusion, as shown in Supplement Table S3 and Figure 2, the FC changes in PDD were much more extended than in PD-MCI, involving practically all functional networks related to global cognition, attention, memory and other domains, with a breakdown of FCs in the DMN, SAN, FPN and BGN as well as the hippocampus, indicating a widespread breakdown not only of the brain networks that are responsible for global cognition and related functions.

## 8. Neurophysiological Studies in PD-CI

Neurophysiological methods offer the advantage of observing oscillatory patterns, whose regional and temporal profiles may elucidate how cognitive changes relate to neuronal mechanisms. Aberrant brain oscillations are a hallmark of PD pathophysiology, and cognitive deficits are thought to be related to altered functional brain connectivity. FC disruptions correlate with cognitive phenotypes in PD-MCI and PDD groups, typically in the α band, and EEG data indicate that FC decreases with the worsening of cognitive performance, and the loss of frontotemporal connectivity may be a promising neuromarker for PD-CI [302].

Both PD-NC and MCI patients had diminished δ and α phase; however, electrophysiological abnormalities were more pronounced in PD-MCI over frontal, central, parietal and temporal locations in almost all frequency bands, which was accompanied by bilateral thalamus, hippocampus and right putaminal atrophy [254]. Visual networks were more severely affected in PD-MCI, which was possibly related to dysfunctional subcortical modulating centers [303].

The evaluation of dynamic FC and the stability of functional networks in PD-MCI compared with early PD without MCI found that the α band as well as the functional network stability (FNS) of the central region, right frontal, parietal, occipital and left temporal lobes were abnormally increased, and the dynamic connectivity fluctuations in these regions were significantly decreased. In the γ band, PD-MCI patients showed decreased FNS in the central, left frontal and right temporal lobes, and there were active dynamic fluctuations in the left frontal, temporal and parietal lobes. These network changes were negatively correlated with cognitive function in early PD [304]. A significant joint effect of interventions on EF and task-based changes for the negative correlation between attention and θ power, a positive correlation between EF and α power, and a significant negative relationship between attention and θ power were found over time [305]. Cognitive deficits were further underlined by alterations at the brain network level, including higher δ and θ activity, lower α and β activity, increased connectivity, and segregated network organization [306].

PD-MCI patients showed altered left and right posterior middle frontal gyrus (PMFG)-based FC in the θ frequency bands. Poorer visuospatial function was associated with higher right PMFG FC under closed eyes and poorer attention function with higher left and right PMFG-based FC, which were independent risk factors for CI [307]. Oscillatory power changes in the cortico-striato-thalamo-cortical circuits (α frequencies in the caudate nucleus and α and θ in the dorsolateral prefrontal cortex) contributed to CI in PD [308].

PD-MCI patients compared to HCs were characterized by more frequent posterior topography of the δ-θ phase-amplitude coupling (PAC) and reversed δ-low frequency α PAC direction, i.e., posterior-to-anterior rather than anterior-to-posterior. This suggested that they showed abnormal neurophysiological oscillatory mechanisms mainly led by δ frequencies underpinning FC from frontal to parietal cortical areas [309].

Both the caudate nucleus and dorsolateral prefrontal cortex displayed decreased β oscillations during memory encoding in individuals with CI suggesting potential dysfunctions in the cortico-striatal circuit. Further analyses revealed differences in the α band in both regions and in θ in the dorsolateral prefrontal cortex, highlighting the involvement of the medial and lateral prefrontal cortex in motivation and cognitive control [310]. Low-frequency mid-frontal neural activity was associated with cognitive dysfunction [311,312,313], while others did not find any relationship between DMN FC and cognition in PD [277], which may be attributed to the vast heterogeneity of PD [281].

Aberrant neurophysiological signals were also associated with speech impairments in PD, in particular in the left inferior frontal cortex, the FC of this region with somatomotor cortices mediating the influence of cognitive decline on speech deficits [314].

Decreased θ, α and β activities were found in both the dorsolateral prefrontal cortex and caudate nucleus during working memory and correlating with cognitive dysfunction in PD patients [308]. The latter was associated with increased response latencies and decreased mid-frontal δ power across all tasks [315]. In general, cognition-related changes in EEG activities were observed over multiple spectral rhythms [316]. PDD patients showed decreased θ frequency in the temporal right-frontal left and temporal right-frontal-right regions while in the α frequency band. The most significant decrease was shown in the occipital right-frontal left and occipital left-frontal right areas. Significant asymmetry was seen in the frontal lobes [317]. There were also significant differences in the θ frequency range between many other areas [318].

PD-NC individuals demonstrated reduced P300 event-related amplitudes compared to HCs; PD-MCI patients had lower P300 amplitudes than both other groups and reduced volumes of the putamen. Both P300 amplitudes and putamen volumes showed moderate associations with EFs [319]. Early PD patients with cognitive deficits exhibited significantly larger P300 and N200 components and smaller amplitude N200 components, indicating impaired neuronal processing [320]. A meta-analysis showed prolonged N200 and P300 latencies in PD non-demented (PD-ND) individuals, compared to HCs, and prolonged P300 latency at the Cz electrode site in PDD patients compared to PD-ND ones [321].

Magnetoencephalography showed higher power in lower frequency bands (δ and θ) along with more severe CI and associated with memory, language, attention, and global cognition. Widespread group differences were found in the β band with significant changes between normal cognition and MCI groups. Moreover, bilateral frontal and left hemispheric regions were affected in the other frequencies as cognitive decline became more pronounced. These data suggested that PD-MCI and PDD are qualitatively distinct cognitive phenotypes, and most neurophysiological changes may occur during the time of this transition [322].

## 9. Involvement of Neuromodulatory Systems in PD-CI

Recent brain imaging studies have provided insight into the dysfunction of multiple neuromodulatory studies that are involved in PD-CI. These were not limited to dopaminergic (DAergic) dysfunction but rather demonstrated the parallel alteration of DAergic, cholinergic and noradrenergic systems [323].

### 9.1. Mapping Dopaminergic Modulation

Striatal and extrastriatal DA dysfunction in early PD [324] was associated with cognitive deficits due to abnormal processing in the cortico-basal ganglia circuit with reduced prefrontal and parietal metabolism [186] in the SAN and medial temporal lobe [325]. Drug-naive PD patients showed associations between striatal dopamine loss, WMHs and cognition [326], whereas no correlation was found between neuropsychological scores and DAT availability in those without MCI [327]. Reduced presynaptic DA uptake in the striatum resulted in abnormal processing in the frontostriatal circuit with reduced prefrontal and parietal metabolism [328], whereas mesocortical DA transmission appeared to be preserved [297]. Selective DA loss in the anterior putamen is a big risk factor for developing PDD [329,330].

The degradation of DAergic afferents to the anterior cingulate cortex played an essential role in cognition, as it is a key node of the SAN [331]. The depletion of dopamine increased signal variability in the SAN, which in turn lead to decreased corticostriatal connectivity [279,280]; this was supported by altered SAN-BGN FC associated with PD-CI.

PD-MCI patients compared to PD-NC ones showed a more widespread degeneration of dopamine terminals in the dorsal caudate nucleus, while other DAergic systems remained relatively preserved. In contrast, PDD patients showed a severe loss of lateral DAergic systems in frontal, temporal and parietal systems [332]. Degeneration of the medial substantia nigra (SN) and nuclei of other ascending pathways caused dysfunction of the striato-subfrontal and mesocortico-limbic loop [333]. Recent multivariate longitudinal analyses confirmed the impact of the dopamine system on long-term CI in PD [334]. MRI radiomics derived from the caudate nucleus were also highly associated with cognitive decline in PD [136].

### 9.2. Cholinergic Modulation

Striatal dopamine release can be triggered not only by the ascending activity of SN DAergic neurons but also by the activity of striatal cholinergic interneurons [335,336]. Cholinergic forebrain atrophy predicted cognitive decline in de novo PD [337], and PD patients with a smaller NBM volume had a 3.5-fold greater risk of developing MCI within 5 years [250]. Early cognitive deficits in PD without dementia were more closely related to structural MRI measures of cholinergic forebrain degeneration than hippocampal degeneration [338]. Early degeneration of the NBM and the nucleus of the vertical limb of the diagonal band of Broca (nvlDBB) and their projections preceded/predicted CI in PD [250,339], whereas sreuctural and microstructural alterations in the entorhinal cortex, amygdala, hippocampus, insula, and thalamus were not predictive of the development of cognitive impairment in PD [340]. In PD-MCI patients, the volume of the NBM was positively correlated with cortical thickness in the bilateral posterior cingulate, parietal, frontal gyri and the left insular region [341].

DTI-based diffusion measures were inferior to standard volumetric assessments for capturing cognition-relevant changes in PD-NC [337]. Volume loss, elevated water content and structural metrics in the cholinergic forebrain and NBM were associated with CI in PD [342,343,344]. The PD-MCI group showed imbalanced associations between different cholinergic basal forebrain nuclei (putamen) and cortical regions (middle frontal gyrus, paracingulate and cingulate gyri), revealing that basal forebrain changes were frequency specific for different cognitive scores [345]. PD-MCI converters had significantly greater mean diffusivity in both NBM tracts compared to PD-NC; trends were identified between the lateral tract mean diffusivity and poorer visuospatial performance, working memory decline, and medial tract mean diffusivity and reduced psychomotor speed [346].

WM lesions in the cholinergic projections from the NBM to the cortex, hippocampus and amygdala were associated with CI over a 5-year period in previous PD-NC [250,347,348]. The loss of basal forebrain cholinergic projections to the hippocampus correlated with memory deficits and conversion to PDD [249,257].

PDD was associated with the selective destruction of corticostriatal resting FC [288]. It further showed a global cortical decrease in 123I-IBVM (vesicular acetylcholinesterase transporter/VAChT) binding in the frontal and posterior cingulate cortex, while reduced IBVM-binding in posterior brain areas was related to cognitive decline [349]. A loss of hippocampal cholinergic fibers occurred in PD-MCI patients, whereas those with PDD were affected by a subsequent increase in αSyn deposition and the dysfunction of both basal forebrain and hippocampal cholinergic systems [339,350].

### 9.3. Noradrenergic Modulation

The locus ceruleus (LC) is a primary source of ascending noradrenergic innervation in the CNS. Its structural integrity can be measured in vivo using neuromelanin-sensitive MRI that was reduced in PD-MCI patients [351,352]. Noradrenaline has been linked to attentional shifting and response inhibition, which were mediated by prefrontal operations [353]. PD patients showed significantly decreased levels of CSF noradrenaline and 3-methoxy-4-hydroxyphenylglycol as well as noradrenaline transporter availability in the hippocampus, indicating significant noradrenergic dysfunction [354].

A severe loss of claustrum dopamine and noradrenaline (serotonin levels unchanged) may interact with the cortico-claustro-cortical information processing mechanisms by passing via the basal ganglia-thalamo-cortical circuits [355]. In vivo neuroimaging studies have shown that acetylcholine and noradrenalin relate not only to gait control but interact closely with cognition. They go beyond the dual-syndrome hypothesis, suggesting that fronto-subcortical noradrenaline and serotonin deficiency are responsible for EF in PD-NC patients. The relationship between posterior cortical cholinergic dysfunction and visuospatial function and semantic fluency as well as the relationship of the noradrenergic LC, serotonergic dorsal raphe nucleus and ventral tegmental area with certain cognitive domains need further investigation [4,356,357,358].

In summary, non-DAergic systems interact closely with the striatal dopamine system, the activity of which is modulated by cholinergic interneurons and other transmitter systems [359]. Neuroimaging and neuropathological studies have demonstrated the role of parallel alteration of cholinergic, noradrenergic and serotonergic systems in cognition in PD.

## 10. Brain Positron Emission Tomography Studies in PD-CI

^18^F-fluorodeoxyglucose PET (FDG-PET) studies in PD-MCI patients showed reduced glucose metabolism in posterior cortical regions, especially in the parietal and cingulate cortex, which were not affected in PD-NC individuals [187,188]. Hypometabolism in parietal, precuneus, hippocampus and occipital lobes was obvious in PD with incident dementia [186,187]. PD-MCI patients exhibited hypometabolism in the frontal and parietal regions compared with PD-NC ones, while it was higher in PDD patients than in those with MCI, mainly in the posterior cortical areas [360,361]. Correlations of reduced metabolism in the areas with event-related potentials confirmed the important role of EF disorders [362]. Patients with atypical brain hypometabolism showed a higher incidence of dementia (60% vs. 3%) and hallucinations (56% vs. 7%) [363].

Decreased glucose metabolism in the left prefrontal cortex and posterior cortex was related to executive and memory dysfunctions, while changes in visuospatial function were associated with hypometabolism in the right and left occipital cortex, suggesting that cognitive function assessment can indirectly reflect the level of glucose metabolism in the relevant brain regions [190]. PD MAPT H1/H1 carriers without dementia exhibited hypometabolism in the frontal cortex, parahippocampal, and cingulate gyrus, in the caudate and globus pallidus, and worse performance in attention than MAPT H1/H2 carriers [364]. In none of these PDD cases was depression/anxiety mentioned, which is also associated with hypometabolism in the frontal cortex [365].

PDD brains showed altered metabolism from several pathways, including glucose and purine metabolism as well as decreases in proline, serine and deoxyguanosine that reflected the progression of αSyn Braak staging [366].

PD-MCI patients exhibited significantly reduced N-acetyl aspartate (NAA), total NAA, choline (Cho), glutathione (GSH), glutamate + glutamine (Glx) and total creatine (tCr), but elevated levels of myo-inositol (Ins) and Ins/tCr ratio as well as reduced NAA/Ins ratio. These findings suggested PD-NC individuals with low NAA and tCr in the posterior cingulate cortex may be at risk of preclinical MCI [367].

^18^F-florbetapir PET showed a higher degree of Aβ uptake in PD patients at higher risk of developing CI [208,368], contributing to memory impairment and a faster rate of cognitive decline [209]. In PD-NC patients, the target regions for Aβ deposition were in the cingulate, middle occipital and middle temporal gyri, while changes in multiple brain regions were correlated to the performance of different cognitive domains [211]. There was a significant negative association between Aβ deposition and language and memory in the PD group [369]. Older PET studies reported an Aβ deposition prevalence of 0.05 in MCI and 0.34 in PDD cases [370], whereas others did not find an association between Aβ deposition and CI [210,371] and no significant differences between PD-MCI, PD-NC and age-matched controls [210,372].

A more recent study detected higher Aβ deposition in the frontal, temporal lobe and prefrontal cortex in PD-MCI [373]. The Aβ-positive group with more aMCI had lower dopamine activities in the left ventral striatum, suggesting AD-related cognitive changes [374,375], although, in general, PDD patients have a lower incidence and severity of Aβ deposition than AD [374,376,377] or DLB patients [378,379]. In patients with PDD, low CSF Aβ-42 level at baseline was not associated with a specific cognitive profile; memory domains and long-delay free recall were not different from those with increased CSF Aβ levels [380].

^18^F-flortaucipir PET showed a gradient of tau binding from PD-NC (none or minimal) via PD-MCI (minimal) and PDD (low/modest) to DLB (intermediate/strong) and finally to AD (highest) [381], tau uptake in PDD being intermediate between PD-ND and AD [213]. Likewise, ^18^F-florzolotau uptake was significantly higher in the cortical regions of PDD patients compared to PD-NC especially in the temporal lobe; uptake in the occipital lobe showed significant correlation with CI [382]. In conclusion, CI in PD is primary linked to biomarkers associated with both AD and PD.

## 11. Neuropathology of PD-MCI

There are few detailed neuropathological studies of PD-MCI. Among 698 autopsy-proven cases of PD, 16 had met the clinical criteria of MCI [383,384]. They included seven naMCI cases (mean age 80 years, 50% brainstem predominant, 31% brainstem–limbic, and 19% neocortical LB stages, with Braak NFT (neurofibrillary tangle) stage 0–4 (mean 2.1), two with neuritic plaques and four with Aβ plaques) and eight aMCI cases (Braak NFT stage 1–4 (mean 2.7), four with neuritic plaques, and three with Aβ plaques). One aMCI multiple domain case, aged 75 years, was LB brainstem type with Braak NFT stage II, few neuritic and many Aβ plaques. One case each also showed mild cerebral amyloid angiopathy (CAA); most of them had a mild lacunar state in the basal ganglia. The neuritic Braak stage in aCMI was higher than in naMCI, mild to moderate neuritic plaques were present in 43%, mild CAA was present in 11%, lacunar state was present in 25%, and old cerebral infarcts were present in 12%. A recent autopsy study of 159 PD cases included 25 MCI (56% aMCI and 44% naMCI with no significant differences in age, gender and disease duration). All naMCI were brainstem–limbic LB stage 3, while in the aMCI group, only 22% were neocortical LB stage 4. Concomitant non-AD tauopathies were present in nine PD-MCI cases, both aging-related tau astrogliopathy (ARTAG) and argyrophilic grains were seen in five cases; two aMCI cases met the pathological criteria of progressive supranuclear palsy. No differences were found in the neuritic plaque stage, total Aβ score, WM rarefication, cerebral infarct volume, CAA score and APOE carrier frequency. There were some differences between the MCI subtypes; aMCI cases had a slightly higher Braak NFT stage, while naMCI-PD cases had an increase in LB pathology [385]. In conclusion, the available autopsy data for PD-MCI revealed a heterogenous picture with the presence of various types of pathological changes similar to MCI in other diseases [386,387], in which the role of other pathologies should be further explored.

## 12. Neuropathology of PDD

The majority of autopsy reports of PDD tried to evaluate the convergence and interactions between basic PD (Lewy) pathology and other cortical proteinopathies (αSyn, tau and Aβ) and their contribution to dementia pathogenesis [33,34,357,388,389,390,391,392,393,394,395] and the role of co-pathologies on cognition [203,389,395,396,397].

The morphological substrate of PDD is heterogenous and includes the following: (1) Lewy body (αSyn) pathology (LBP) in cortical, limbic and subcortical/brainstem structures, (2) AD-related neuropathological changes (ADNC)—diffuse Aβ and neuritic plaques, NFTs and CAA, and (3) a variable combination of these pathologies that has been differently related to the severity of CI [357,390,394,395,398]. Limbic and neocortical LBP were significantly higher in PDD than in MCI, CI often correlating with the severity of LBP in the frontal cortex, hippocampus and periamygdaloid cortex, causing a disruption of the limbic loop similar to that in AD [399]. LBP severity in the CA 2/3 region of the hippocampus was related to episodic memory loss [400]. Mice injected with αSyn fibrils developed progressive spatial learning and memory deficits associated with hippocampal and cortical αSyn pathology with extensive neurodegeneration of the CA 2/3 hippocampal subfield at 6 month post-injection and, therefore, it can be used as a model for Lewy-related cognitive dysfunction [401]. However, recent studies revealed that PD-MCI patients had smaller bilateral CA1 volumes than PD-NC, although the same was observed in a de novo PD cohort, providing evidence that CA1 changes may be the earliest indicator of emerging CI [402]. LBP in PDD was higher in subcortical regions than in PD-NC, and its density in the amygdala and hippocampus correlated with dementia severity [403,404]. In a large autopsy study, the severity of neocortical LBP correlated best with dementia severity [405] and more rapid decline in all cognitive domains, whereas limbic LBP was associated with a more rapid decline in visuospatial skills, which was not modified by coexistent ADNC [406]. In another autopsy study, combined LBP and ADNC showed worse deficits in the executive/visuospatial domain than LBP only when LBs were confined to the brainstem, but there were no differences when LBs extended to the limbic or cerebral cortical regions. That means that the impact of co-occurring ADNC on antemortem cognitive deficits varies not only by domain but also on the pathological stage of LBP [407].

It should be considered, however, that not all patients with cortical LBP may develop dementia [408,409] despite its more severe increase in inferior frontal gyrus in PDD [410]. The association between cortical LBP and dementia was challenged by some studies that reported that 15–44.7% of PD-NC patients were associated with severe neocortical LB burden [390,398,409,411], while a small study described PD cases without dementia despite considerable cortical and limbic LBP [408]. On the other hand, in 14.7% of PDD cases, LBP was confined to the brainstem without cortical involvement [411]. A large autopsy study showed that in PD patients, the Braak LB stage was associated with dementia, whereas the severity of ADNC and small vessel pathology did not; thus, neocortical LBP very often, but not always, paralleled dementia in PD, while it also appeared in PD patients without dementia [394]. In a recent personal autopsy study of 210 PD cases, 47.6% with dementia, PDD cases were significantly older at death than PD-NC ones (mean 83.9 vs. 77.9 years, *p* < 0.05) and had a shorter disease duration (mean 9.2 vs. 14.5 years). Their Braak LB score and NFT stage were significantly higher (mean 4.2 vs. 4.1, and 4.4 vs. 2.3, *p* < 0.01), as were the Aβ phase (mean 3.0 vs. 1.8) and CAA frequency and intensity (50% vs. 24%, and 0.7 vs. 0.3, all *p* < 0.01) [34].

This and other autopsy studies have confirmed the co-existence of αSyn, Aβ and tau pathologies in PD with CI and its impact for dementia development [357,388,398,412,413]. ADNC, severe enough for a pathological diagnosis of AD, was present in about 10% of PD-ND patients, 35% of PD-MCI patients and over 50% of PDD patients [28,395,405]. In large autopsy series, around 50% of PD cases showed Braak LB stages 4–6 and NFT stages 4–6 [34,390], while others suggested a significant relationship between cortical LBP and severity of dementia [208,395,414]. Four large autopsy studies of PD showed consistent results: while co-morbid ADNC was present in 19–31.5% of total PD cases, the rate of co-morbid ADNC in PDD ranged from 21.5% to 89.4% [405,413,415], tau pathology being more severe in the prefrontal than in the temporal cortex [416]. Some studies indicated that dementia in PD was related to co-morbid ADNC, the spread of tau being similar to that in typical AD, although in some cases, neocortical tau was variable and the medial temporal lobe was relatively spared [417,418]. A recent meta-analysis stated that the four clinical core features were more common in PD-MCI than in AD-MCI/stable MCI [419].

Despite some differences between study groups, Aβ deposition was not specifically related to dementia in PD, but it may be linked with more rapid cognitive deterioration and earlier mortality [404,420,421,422,423]. On the other hand, significant Aβ burden was found in 15–45.4% of PD cases without CI [398,405,409], while other studies found considerable αSyn, Aβ and tau pathologies in elderly PD patients without CI [424], which could be explained by there being a higher cognitive reserve in these patients [425].

In conclusion, there is increasing evidence for a synergistic effect of αSyn (Lewy) and tau (AD) pathologies as the main driver of cognitive decline/dementia in PD, the relative impact of which is under discussion. A recent study on the disease-specific patterns of αSyn destabilization revealed differences of the cytosolic unfolded, monomeric form of αSyn (αSU) and the cytosolic helically folded, multimeric form (αSH) equilibrium comparing demented and cognitively intact PD patients [426]. These data indicate that the region-specific susceptibility of LBP is a major component for the development of CI in PD but not always, which was confirmed by recent autopsy studies [394].

## 13. Cognitive Reserve and Resilience in PD

Long-time life experiences, such as education, occupational attainment, leisure activities, modifications of lifestyle, and bilinguism, have been considered proxies of cognitive reserve (CR), which is considered as a modulator of a more favorable cognitive trajectory, less disability and better quality of life. Increased physical activity has been shown to attenuate APOE ε4-related vulnerability to early cognitive decline in patients with PD. This protective effect did not appear to be mediated by striatal DAergic function [427].

It was hypothesized that higher CR would correlate with better performance and is associated with a mild increase in performance with regard to verbal learning, wording memory, and problems in understanding the pragmatic aspects of language experience by patients with PD [428]. There is strong evidence that higher CR was associated with a mild increase in performance with regard to verbal fluency, learning and recognition, working memory and visuoconstructive ability. This suggested that CR moderates the effects of brain morphology and functions on cognition in PD. Multiple CR-related processes may offer resilience against functional decline, among which CR refers to the adaptability of cognitive processes. Network analyses demonstrated that relative to PD patients experiencing cognitive decline, the FPN in cognitively stable individuals was significantly more resilient to network perturbation. This suggested that the topological robustness of the FPN is associated with less cognitive decline in PD [429]. Compared to individuals with high CR, those with low CR had reduced posterior DMN FC in the anterior cingulate gyrus and basal ganglia as well as bilateral reduced connectivity in fronto-parietal regions within the prefrontal networks, while hyper-connectivity was detected within medial prefrontal regions. This suggests that CR may exert a modulatory effect on FC in the basal ganglia and executive–attentional fronto-parietal networks in PD patients with low CR. Attentional control networks seem to be downregulated, whereas a higher recruitment of medial frontal regions suggests compensation via upregulation mechanisms that may contribute to maintaining cognitive functioning when posterior control mechanisms are reduced [430].

Resilience is defined as the process of adapting well in the face of adversary, threats, trauma, tragedy or significant sources of stress. High levels of social support were associated with increased resilience, which in turn was associated with reduced mental health symptoms [431], and correlated with less disability and better quality of life but not with PD severity [432]. Exercise regulates the neuroendocrine system, whose primary role is to respond to stress, maintain homeostasis and improve resilience in aging and neurodegeneration. Exercise in PD has been shown to exert some cognitive and behavioral-induced control over their disease [433].

Stress susceptibility and resilience were mediated by the noradrenergic regulation of DAergic neurons [434]. Recent studies have shown that a genetic resilience score can modify the penetrance of PD risk factors and thereby protect individuals carrying high-risk genetic burden for developing PD. Although no single locus reached genome-wide significance, multimarker analysis of genomic annotation (MAGMA) gene-based analyses nominated TBCA as a putative gene associated with resilience in PD [435]. LRRK2-PD patients showed a significantly higher basal forebrain volume compared to iPD and developed no cognitive changes over a 4-year follow-up period, potentially reflecting a compensatory cholinergic state that could prevent cognitive decline [436], which also might partially be attributed to a slower increase in NfL levels [437]. The LRRK2-G2019S mutation that causes late-onset PD affects striatal neuron function and alters synaptic plasticity, thus promoting resilience to chronic social stress and supporting cognitive function [438]. ATG8 proteins, co-factors for DAergic neuronal transcriptional control, have been proposed as autophagy gene transcriptional co-factors for stress protection of ventral midbrain DAergic neurons and neuronal resilience in PD [439].

In conclusion, CR is suggested to play a role in the presentation of CI, but it is not protective against general cognitive decline, although there is evidence that it protected several cognitive domains from further decline. CR has been documented to modulate the extent of cognitive decline or to protect language fluency and other cognitive domains from further decline. Likewise, resilience is correlated with less disability and cognitive decline, although it shows no correlation with PD severity.

## 14. Impact of Other Co-Pathologies in Cognition in PD

A number of other neuropathologies, particularly those associated with age, can influence the course and the severity of CI in PD. In addition to the high prevalence of ADNC, TAR DNA-binding protein 43 (TDP-43) and cerebrovascular pathologies, due to their high prevalence and clinical impact on PD patients, show both cross-sectional and longitudinal clinical importance [440]. The extent and severity of cerebral small vessel disease (CSVD) has considerable impact on the severity of CI. A meta-analysis of its influence on cognitive function in PD showed different effects—most on EF, memory and overall cognitive function [441], whereas others found no correlation between CSVD pathology and dementia [442,443]. Lacunes were independently associated with worse attention and processing speed and may accelerate the course of PD [444]. Increased perivascular space in the basal ganglia and WMH was associated with cognitive decline in PD [203,445], as were cerebral microbleeds (CMB), which were both related to hypertension [397]. CMBs were significantly associated with PDD [396,443], while others associated PDD with amyloid-related CMBs and reduced hippocampal volume [446]. The association of severe CAA with PDD has been reported repeatedly [34,398,405], whereas, according to others, cerebrovascular disease and TDP-43 pathologies had no definite impact on the development of PDD [395]. Argyrophilic grain disease, an age-related 4-R tauopathy affecting the medial temporal lobe, although a rare co-pathology, may be another important factor inducing dementia in PD [447], whereas others did not find such a combination [25,405]. Most of these and other age-related co-pathologies make it difficult to disentangle their individual contribution to cognitive decline [398,412,417], but in general, a close cooperation of various neuropathologies is responsible for the development and progression of cognitive and other clinical deficits in PD [389,448].

## 15. Conclusions and Outlook

PD, a common and heterogenous neurodegenerative disease, is characterized by a combination of motor and non-motor symptoms including CI involving multiple cognitive domains. The morphological and molecular/biochemical basis of CI is heterogenous, and modern neuroimaging studies revealed widespread changes in cerebral GM and WM, involving multiple brain areas and causing the disruption of FC for many critical neuronal networks involved in cognitive, attentional, memory, and behavioral functions. PD patients exhibiting “AD-like” patterns of brain atrophy are at greater risk of developing cognitive decline than PD-NC individuals and HCs [449]. The majority of autopsy-based studies support a strong association between corticolimbic LBP and ADNC resulting from the complex interaction of αSyn, Aβ, tau and other pathological proteins (like PTD-43) resulting in the complex interplay of progressive motor and cognitive decline in PD. It has been proposed that the development of cognitive decline prior to or simultaneous with the manifestation of motor symptoms should be included in the diagnostic procedures of PD [450,451]. The prospective assessment and validation of PD-CI and a deeper understanding of the interaction of multiple pathogenic factors will be achieved by modern fluid biomarkers (reduced αSyn and Aβ-42 levels, increased p-tau 181, glial fibrillary acidic protein/GFAP/and NfL) as well as in post-mortem brain tissue [452,453,454,455,456,457,458] that will also enable a differentiation between PD-CI and AD. In order to elucidate these and other open questions, a combined assessment of in vivo neuroimaging and liquid biomarkers in multicentric longitudinal clinico-pathological studies is warranted that also may contribute to the development of meaningful disease-modifying therapies and preventive measures to slow the progression of PD and associated cognitive deterioration.

## Supplementary Materials

The following supporting information can be downloaded at https://www.mdpi.com/article/10.3390/ijms25010498/s1. References [42,55,80,140,152,153,154,155,156,157,158,159,160,161,162,163,164,165,166,167,168,169,170,171,172,173,174,175,176,177,178,179,180,181,182,183,184,185,186,187,188,189,190,191,192,193,194,195,196,197,198,199,200,201,202,203,204,205,206,207,208,209,210,211,212,213,214,215,216,218,219,221,224,229,237,243,244,245,246,247,248,249,250,251,252,253,254,255,256,257,258,259,260,262,264,265,266,267,269,275,277,281,282,283,284,285,286,287,288,290,291,293,296,310,328,333,346,355,374,376,377,381,382,459] are cited in the supplementary materials.

## Abbreviations

AD	Alzheimer disease
ADNC	AD-related neuropathological changes
aMCI	amnestic mild cognitive impairment
Aβ	amyloid-β
αSyn	α-synuclein
BGN	basal ganglia network
CAA	cerebral amyloid angiopathy
CI	cognitive impairment
CN	cerebellar network
CR	cognitive reserve
CSF	cerebrospinal fluid
DLB	dementia with Lewy bodies
DMN	default mode network
DTI	diffusion tensor imaging
EF	executive function
FA	fractional anisotrophy
FC	functional connectivity
FOG	freezing of gait
FPN	frontoparietal network
GBA	glucocerebrosidase
GDNF	glial cell line-derived neurotrophic factor
GM	gray matter
GMV	gray matter volume
HCs	healthy controls
LBs	Lewy bodies
MCI	mild cognitive impairment
MDS	Movement Disorder Society
naMCI	non-amnestic mild cognitive impairment
NBM	nucleus basalis of Meynert
NfL	neurofilament light
NFT	neurofibrillary tangle
PD	Parkinson disease
PDD	Parkinson disease dementia
PD-MCI	Parkinson disease with mild cognitive impairment
PD-NC	Parkinson disease with normal cognition
PD-ND	Parkinson disease non-demented
RBD	REM sleep behavior disorder
rsfMRI	resting-state functional MRI
SAN	salience network
SCD	subtle cognitive decline
SMN	sensorimotor network
WM	white matter
WMH	white matter hyperintensity
