# Supplementary material for: Pathobiology of Cognitive Impairment in Parkinson Disease: Challenges and Outlooks
Source: Int J Mol Sci. 2023 Dec 29;25(1):498. doi: 10.3390/ijms25010498 (PMC10778722; doi:10.3390/ijms25010498)
Supplement: Supplementary file 1 [file ijms-25-00498-s001.zip › ijms-2762227-supplementary.pdf]

Table S1 (SUPPLEMENT): Major neuroimaging finding in Parkinson disease with mild cognitive impairment (PD-MCI)

	**References**
General	
Mild diffuse brain atrophy	[152,153]
Enlargement bilateral temporal and lateral ventricles<br>Enlargement third ventricle	[154,155]
Gray matter atrophy	
Prefrontal, limbic lobes, left temporal gyrus	[156]
Anterior cingulate and right parietal lobe	[55,157]
Orbitofrontal regions, left superior lobule, limbic, fronto-parietal regions	[158]
Parieto-temporal cortex	[159]
Frontal, temporal, parietal and occipital cortex, striatum	[160]
Frontal and limbic lobes	[161]
Prefrontal, medial and lateral temporal cortex	[162]
Superior frontal cortex, bilateral entorhinal cortex	[163]
Bilateral dorsolateral prefrontal cortex, left insula, angular gyrus, cingulate and right supramarginal gyrus	[164]
Frontotemporal cortex, parietal and occipital lobes	[165]
Temporal and parietal cortex, amygdala, hippocampus, putamen, cerebellum	[166]
Medial and superior frontal, inferior temporal, cingulate and supramarginal gyri	[167]
Left inferior and orbital frontal gyrus, left anteriorinsula	[168]
Subiculum, presubiculum, left parasubiculum, hippocampal CA1 and fimbria	[169-171]
Subcortical structures: caudate nucleus, putamen, thalamus, amygdala, nucleus accumbens, NBM, presubiculum	[172,173]
White matter lesions	
Higher WM lesion burden periventricular and deep WM	[174,175]
High WMH burden occipital WM	[176]
WMH prefrontal and temporal lobe	[177]
Involvement corpus callosum, cingulum, frontal, interhemispheric WM	[178]
FA WM corpus callosum	[179]
WM microstructure lesions	[180]
Decreased FA bilateral frontal and temporal lobes, corpus callosum, cingulum, fornix, longitudinal and fronto-occipital fasciles, bilateral parieto-occipital tract	[181]

Lower density prefrontal region, cingulum bundle, thalamofrontal tracts	[182]
Microstructural changes in anterior olfactory structures	[183]
FA fornix	[161]
Decreased FA in fronto-occipital fascicles	[184]
Decreased FA in superior longitudinal fascicles	[185]
Reduced glucose metabolism	
Parietal, cingulate cortex, precuneus, hippocamous, occipital lobes	[186-188]
Hypometabolism exceeding atrophy in angular gyrus, anterior frontal, orbital and occipital lobe, precentral and supplementary motor areas	[189]
Frontal and posterior cortex, bilateral occipital lobe	[190]
Brain network dysfunctions	
Loss of FC in SAN without structural changes	[191]
Dysfunction of sensorimotor, executive and neurocognitive networks	[192]
Reduced FC between striatal network, DMN, central executive and SAN	[192]
Increased FC betwen left hippocampus and right cerebellar hemisphere	[192]
Reduced FC within DMN between hippocampus and inferior frontal cortex, posterior cingulate cortex and posterior parietal lobuls, anterior temporal lobe and inferior frontal gyrus, middle frontal and middle temporal gyrus, between DMN and precentral middle temporal gyrus, insula, anterior parietal lobuse and middle frontal cortex	[193]
Reduced FC between bilateral frontoparietal network, ventrolateral and dorsolateral prefrontal cortex, dorsolateral prefrontal cortex and inferior frontal gyrus	[194]
Abnormal intrinsic FC within DMN and SAN, decoupling between left and right FPN	[195]
Decreased FC in DMN and frontoparietal network	[196,197]
Decreased FC in sensorimotor network and DMN networks	[198]
Disruption DMN and cerebellar network	[197]
Reduced FC multi-connect networks in bilatera orbitofrontal lobe	[199]
Reduced FC between mediodorsal thalamus and paracingulate cortex<br>Increased FC mediodorsal thalamus and posterior cingulate cortex	[200]
Disrupted network between bilateral superior medial frontal cortex and anterior/middle cingulate cortex	[201]
Decreased FC between right caudate head and anterior cingulate cortex, precuneus and left supramarginal gyrus	[192]
Increased FC between caudate head to left hippocampus and right cerebellum	[201]
Disrupted FC between frontal and posterior cortical regions	[202]

Decreased FC in SMN and executive processing networks	[164]
Decreased FC between frontostriatal and posterior cortical areas	[160]
Disrupted FC between frontal cortex and posterior cortical regions	[80,203]
Reduced FC between right frontal and bilateral parietal areas<br>Decreased FC between substantia innominata and frontal area	[204]
Disrupted FC between frontoparietal-visual-sensorimotor and subcortical networks	[205]
Increased amplitude of low-frequency intrinsic fluctuations and regional homogeneity in the DMN	[206,207]
Other changes	
High free water fraction in caudate nucleus, bilateral NBM	[172]
Mild cortical Aβ binding in cingulate and middle temporal gyri, not significantly different from PD with normal cognition and age-matched healthy controls	[208-211]
Rare and minimal cortical tau deposition	[212-214]
Reduced neuromelanin-sensitive PET scan signal in substantia niga and locus ceruleus	[215]

DMN: default mode network; FA: fractional anisotrophy; FC: functional connectivity; FPN: frontoparietal network; SAN: salience network;

Table S2 (SUPPLEMENT): Neuroimaging findings (a) in PD-MCI converters vs. non-converters; (b) PDD converters vs. non-converters, (c) PDD converters vs. PD-MCI non-converters

**Type of lesion**	**Reference**
(a)	
GM atrophy frontal and temporal lobe, hippocampus, thalamus, nucleus accumbens	[237]
Lower GM thickness in prefrontal, insular, caudate nucleus, frontotemporal cortex	[80]
Cortical thinning frontal and temporal medial occipital lobes	[221]
GM atrophy frontal and temporal lobes	[242]
Cortical thinning medial and superior frontal, inferior temporal, bilateral cingulate and supramarginal gyri	[167]
Cortical thinning bilateral frontal, insula, left temporal areas	[243]
Cortical prefrontal, medial and lateral temporal areas	[162]
Atrophy thalamus, striatum,hippocampal subfield CA 2/3 , right presubiculum	[170,171]
Lower GM density left prefrontal and insular cortex, bilateral caudate nucleus	[140]
(b)	
Larger WMH volume and higher hyperintensity WM frontal lobe	[244]
Cortical thinning left superior temporal gyrus,right insula and fusiform gyrus, bilateral fusiform gyri, decreased functional connectivity between these affected regions	[219]
Disrupted WM connectivity frontal and posterior cortical regions	[202]
(c)	
GM atrophy left cingulate and right inferior temporal gyrus, volume reduction substantia innominata	[245]
GM atrophy prefrontal. area, bilateral caudate nucleus, insula, olfactory cortex,	[246]
Cortical thinning prefrontal, medial and lateral temporal regions	[80,162]
Volume reduction global WM; diffusion-based free water measure in hippocampus and dorsolateral thalamus	[247]

PD-MCI: Parkinson disease with mild cognitive impairment; PDD: Parkinson disease dementia; GM: gray matter; WM: white matter; WMH: white matter hyperintensity

Table S3 (SUPPLEMENT): Neuroimaging changes in Parkinson disease dementia (PDD) vs. Parkinson disease non-demented (PD-ND)

**Type of lesion**	**References**
General	
Increased whole brain atrophy and enlargement of ventricular system	[166]
Gray matter	
Atrophy of temporo-parietal region	[42,224]
Atrophy anterior temporal, dorsolateral prefrontal, posterior cingulate, temporal fusiform, occipitotemporal cortex and hippocampus	[249]
Atrophy bilateral temporal lobe, hippocamous, right cuneus and precuneus, left inferior frontal gyrus and left insula	[250]
Atrophy inferior frontal and superior temporal lobe, anterior cingulate cortex	[251]
Atrophy bilateral insula and hippocampus	[164]
Atrophy bilateral frontal lobe	[252]
Atrophy basal forebrain	[253,254]
GM loss bilateral medial temporal lobe and striatum	[255]
Decreased volume of claustrum	[256,257]
Lower hippocampal volume	[258]
Abnormal diffusvity frontal lob and hippocampus	[259,260]
Free water in dorsomedial thalamus, nucleus basalis Meynert	[247]
Hypometabolism precentral and supplementary motor area, temporal lobe, and hippocampus	[189]
Hypometabolism left prefrontal cortex, bilateral occipital cortex, hippocampus	[190]
Volume decrease in cuneus, lingual and cingulate gyrus, superior parietal lobe, middle frontal gyrus	[261]
Volume decrease right lingual, superior temporal, insula, bilateral fusiform area	[219]
White matter	
Higher white matter hyperintensity burden in deep central white matter	[229,262]

Thinning microstructural lesions corpus callosum	[218,263]
Decreased FA and diffusivity increase in bilateral cingulate tract	[264,265]
Same changes in genu corpus callosum and hippocampus	[266]
Connectivity changes	
Dysfunction of salience network, loss of connection anterior insular and anterior cingulate cortex	[267]
Dysfunction of resting-state FC in left superior temporal, bilateral fusiform gyrus , bilateral fusiform gyrus	[219]
Disorder cognitive cortico-striato-thalamo-cortical circuit	[268-270]
Decreased FC in sensori-motor networks	[271]
Disorder network bilateral hippocampus, left putamen, bilateral anterior temporal poles	[216,272,273]
Reduced FC in dorsal insular cortex	[274]
FC loss between anterior insular and anterior cingulate cortex (SAN)	[267]
FC loss between two DMN systems (left-to-right hippocampus)	[275]
FC loss medial prefrontal cortex to posterior cingulate cortex (SAN)	[275]
FC loss frontal-temporal network right to left hemisphere	[266,276]
Reduced FC between deep gray matter regions and FPN	[277]
Reduced FC deep gray matter regions - frontoparietal network; disrupted WM FC in frontal and posterior cortical region	[202]
Decreased claustral network	[278]
Destruction of striatal resting FC	[270]
Decreased FC postcentral sensorimotor cortex and supplementary motor area, and between claustrum and cingulate/supramarginal gyrus and insular cortex	[279]
Dysfunction of corticostriatal FC	[270,280]
Reduced intra-FPN FC, dysfunction SAN-BGN and SAN-DMN	[281]
Reduced FC between SAN and DMN	[191,282]
Dysfunction FC SAN-BGN and other networks	[281,283]
Others	
Severe loss of lateral dopaminergic frontal-temporal-parietal system	[284]

Loss of dopamine/noradrenalin basal ganglia-thalamo-cortical circuit	[285]
WM lesion of cholinergic projections from nucleus basalis of Meynert to frontal cortex, hippocampus and amygdala	[286]
Hypometabolism in frontoparietal, occipital lobes, precuneus and hippocampus	[186,187]
Frequent moderate to severe cortical Aβ deposition	[287-289]
Frequent moderate to severe tau deposition in cerebral cortex	[290,291]

BGN: basal ganglia network; DMN: default mode network; FC: functional connectivity; FPN: frontoparietal network; SAN: salience network
